# The association of dairy intake of children and adolescents with different food and nutrient intakes in the Netherlands

**DOI:** 10.1186/s12887-015-0524-3

**Published:** 2016-01-09

**Authors:** Marjo J. E. Campmans-Kuijpers, Cecile Singh-Povel, Jan Steijns, Joline W. J. Beulens

**Affiliations:** Julius Center for Health Sciences and Primary Care, University Medical Center Utrecht, P.O. Box 85500, 3508 GA Utrecht, The Netherlands; FrieslandCampina, Amersfoort, The Netherlands

**Keywords:** Dairy, Milk, Food-intake, Children, Adolescents

## Abstract

**Background:**

Dairy products are nutrient-rich foods that may contribute to adequate nutrient intakes. However, dairy intake might also be associated with other food sources that influence nutrient intakes. Therefore, we studied the association of dairy, milk and cheese intake with intake of foods and nutrients from (non)dairy sources.

**Methods:**

Dietary intake was assessed from 2007 to 2010 through two non-consecutive 24-h dietary recalls in 1007 children (7–13 years) and 706 adolescents (14–18 years). Participants were divided into non-consumers of a particular dairy product and tertiles according to their dairy intake (lowest, medium and highest intake). P for trend was calculated by linear regression over the median intakes of non-consumers and the tertiles for dairy, milk and cheese.

**Results:**

In children, higher dairy consumption was associated with higher intakes of fruits (54.8 g ± 22.3; *p* < 0.0001), vegetables (25.0 g ± 14.6; *p* = 0.001) and cereals (18.5 g ± 20.7; *p* = 0.01) and with lower consumption of non-alcoholic beverages (−281 g ± 101; *p* = 0.01): soft drinks (−159 g ± 28.2; *p* < 0.0001) and fruit juices (−40.5 ± 14.8; *p* = 0.01). Results were comparable for milk consumption. In adolescents, similar results were found for milk and dairy consumption, except for the associations with higher fruits and vegetable intake.

In children and adolescents, higher cheese consumption was associated with higher vegetable and non-alcoholic beverages consumption; and lower meat consumption (−7.8 g ± 4.8; *p* = 0.05) in children. Higher cheese consumption was also associated with higher intakes of saturated fat (8.5 g ± 0.9), trans-fatty acids (0.48 g ± 0.06), sodium (614 mg ± 59.3) and several vitamins and minerals .

**Conclusions:**

Higher milk and dairy consumption were associated with lower non-alcoholic beverages consumption, and higher cereal, fruit and vegetable consumption in children, which was also reflected in the nutrient intakes. These findings confirm that the consumption of milk and dairy products might be a marker for healthier eating habits.

**Electronic supplementary material:**

The online version of this article (doi:10.1186/s12887-015-0524-3) contains supplementary material, which is available to authorized users.

## Background

Dairy products are nutrient-rich foods [1], which remain an important source of micronutrients like calcium, vitamin B2 and B12 [2]. In addition, dairy products provide children with energy, high-quality protein, and essential and nonessential fatty acids.

However, dairy products, and especially cheese and high-fat dairy products, may also contribute to an excess intake of energy, sodium, saturated fatty acids (SFA) and trans-fatty acids (TFA) [2]. Despite its contribution to energy intake, recent meta-analyses showed that high dairy intake was associated with lower adiposity in adolescents, while in younger children no association was found, although heterogeneity of the studies was high [3]. A review on dairy intakes in children and adolescents showed that dairy consumption was not or inversely associated with incidence of dental caries, and hypertension; and positively associated with linear growth and bone health during childhood [2].

Nonetheless, in recent decades, the consumption of milk and dairy products by children and adolescents has waned, with a substantial proportion of youth failing to meet intake recommendations [2]. Whereas most studies found dairy consumption declines further with increasing age, particularly throughout adolescence [2, 4, 5], a British study found no difference in milk-based dairy consumption between middle-childhood (9 to 11 years) and adolescence (15 to 18 years) [6].

In the Netherlands, children aged 4 to 8 years are recommended to consume 400 ml of milk and 10 gram of cheese, whereas for adolescents 600 ml of milk and 20 gram cheese is recommended, both preferably low fat [7]. These recommendations are comparable to other developed countries, where children under the age of 9 years are recommended to use approximately 500 ml dairy products and adolescents > 600 ml dairy per day [2].

In the Dutch National Food Consumption Survey 2007–2010 Dutch children and adolescents consumed insufficient fruit, vegetables, fish and fibre and too much SFAs [8]. Furthermore, the intakes of vitamin A, vitamin C, vitamin E, calcium, magnesium, potassium and zinc were below the recommended amounts for certain children, but without health effects [8]. Increased dairy intake could thus contribute to the adequate intakes of nutrients in Dutch children and adolescents.

However, the contribution of dairy to adequate nutrient intakes also depend on replacement of dairy by other food products in the diet. We are aware of only one study that addressed the relation of dairy consumption with intakes of other food groups in 8 to 10 year old children [9]. This study showed that a low milk intake was associated with higher intake of sugar sweetened beverages. A higher dairy intake was also associated with higher consumption of foods from the bread and cereal group and lower consumption from the meat and alternatives group (including fish, eggs, nuts and seeds). The consumption of milk and dairy products might thus be marker for healthier eating habits. Whether intake of dairy products is associated with intakes of other food sources in diet of adolescents has not been investigated to date. It is similarly unknown how such associations are reflected in nutrient intakes from dairy and non-dairy sources.

The aim of the present research is to study whether milk and dairy products are associated with the intakes of other food products in the diet and whether this is associated with a different nutrient intake from non-dairy products.

## Methods

### Study population

For this study, data from the Dutch National Food Consumption Survey 2007–2010 were used [8]. The survey was conducted among 3819 children and adults aged 7 to 69 years. The population was divided into six age categories: 7 to 8 years; 9 to 13 years; 14 to 18 years; 19 to 30 years; 31 to 50 years; and 51 to 69 years. For the current study we included the children 7–13 years (*N* = 1007; response rate 74 %) and adolescents 14–18 years (*N* = 706; response rate 62 %) of this sample. The study population were representative of the general Dutch population according to the levels of education, region, and urbanisation. Recalls were almost equally spread during the week (Saturdays were underrepresented) and during the year (winter was slightly overrepresented). Furthermore, children were overrepresented in the study population. To adjust for these small deviations in socio-demographic characteristics and imbalances in season and the day combination of both consumption days a weighting factor was used. Permission to use the data from the Dutch National Food Consumption Survey 2007–2010 was obtained from National Institute for Public Health and the Environment. Ethical approval and informed consent were deemed unnecessary according to the Dutch legislation [10].

### Dietary assessment

The data were collected through two non-consecutive 24-h dietary recalls (using the computer directed interview program EPIC-soft) [11, 12]. Trained dieticians used a multiple pass approach [11–13]. Children aged 7 to 15 years were interviewed face to face by a dietician with at least one of the parents present during home visits. Participants over the age of 15 were interviewed by telephone, at dates and times unannounced to the participants. Each person was interviewed twice with an interval of 2 to 6 weeks. The recalls were spread equally over all days of the week and the four seasons. Interview days were not planned on holidays. The survey consisted of a description of foods by a further specification of the foods using facets and descriptors such as preparation method and fat content. Portion sizes of foods and meals were quantified in several ways: by means of quantities shown by photos, or in household measures, standard units, by weight and/or by volume. Further, the food frequencies of the intakes of dietary supplements, distinguishing between winter time and during the rest of the year, were asked.

### Dairy intake

We analyzed overall dairy intake, but also the subcategories of milk and cheese intake, because cheese and milk are used differently in the Dutch diet. Therefore, the intakes of dairy products were divided into three categories: 1. Dairy products (the overall category) 2. Milk and 3. Cheese. Dairy products included milk, milk beverages, yogurt, fromage blanc, petits suisses, cheese products, and milk based desserts. Milk contained only milk and buttermilk. Cheese contained cheese products (including fresh cheeses). The mean intake over the two registered days were calculated. Per age category, participants were divided into non-consumers of a particular dairy product and three tertiles according to their dairy intake. Non-dairy consumers were those participants who did not consume any dairy products in the two days of the dietary recalls.

### Food groups

In this study we used food groups based on EPIC-soft classification [12]. We studied 17 food groups and presented the results of the 13 main food groups, including: 1. Potatoes, 2. Vegetables, 3. Fruits, 4. Dairy products, 5. Cereals, 6. Meats, 7. Fish, 8. Eggs, 9. Fats, 10. Sugar_confectionary, 11. Cakes, 12. Non-alcoholic beverages, and 13. Alcoholic beverages.

The food group ‘fruits’ included fruits, nuts and olives; the food group term ‘fats’ was used for fats like margarine and oil. The term ‘soft drinks’ included carbonated drinks, soft drinks, isotonic drinks and diluted syrups. Further, we used ‘fruit juice’ as a generic term for fruit and vegetable juices and chocolate spread as a generic term for chocolate spread, flakes and confetti. Since cheese might be replaced with confectionary (sugar, honey or jam) on bread, these intakes were calculated for the cheese category.

### Measurement on non-dietary factors

Three age-specific general questionnaires were used to collect characteristics that are relevant on the level of the individual person (e.g. age and sex), the properties on the household (e.g. size and income) and lifestyle characteristics of the participant: the activities, general diet characteristics, consumption frequency of certain specific foods, use of dietary supplements (per season) and so on [8].

Since all participants were under the age of 19, the educational level concerned the head of household and was categorized into three categories; low (primary school, lower vocational, low or intermediate general education); middle (intermediate vocational education and higher general education); high (higher vocational education and university) and a category for incomplete information.

Squash (Short Questionnaire to Assess Health enhancing physical activity) questionnaires were used for adolescents to obtain information on physical activity [14]. For the younger children, questions on activities relevant for this age group (like watching television, computer time, sports at school, walking or cycling to school, sport club activities and playing outdoors) were used. Physical activity up to 5 h/week was inactive; >5 h/week was active.

Tobacco use was gathered through the general questionnaire, but not for children. The information on smoking for adolescents was divided into three categories: current smoking of at least one cigarette, cigar or pipe a day, former smokers and never-smokers.

Body weight and height were reported (not measured) to an accuracy of 0.1 kg and 0.5 cm respectively. Body mass index (BMI) was calculated based on the average body weight and height of both interview days. The basal metabolism rate (BMR) was calculated based on sex, weight, height and age according to the Harris-Bennedict formulas [15]. The energy intake BMR ratio was calculated by dividing the mean energy intake by the BMR.

### Statistical analyses

Mean intakes of dairy products were calculated by dividing the intake over the two registered days by two. To study the trends over the intakes of non-consumers and low- and high consumers we divided the participants in non-consumers and tertiles of respectively dairy, milk and cheese intakes. The following items were analyzed for each dairy category: intake of all nutrients, nutrient intake from dairy sources and the nutrient intake from non-dairy sources, the intakes of food groups, specific dairy products and beverages. Estimates (± SE) and P for trend were calculated by linear regression over the median intakes of non-consumers and the tertiles. To generalize the results to the general population, weighting factors to adjust demographic properties, season and the day combination of both consumption days were used as a weighting variable [16]. For this variable the day at which the recall applied to was classified as either weekday (Mon-Tue-Wed-Thu) or as weekend day (Fri-Sat-Sun). In a secondary analysis, these differences were adjusted for energy intake, since a higher dairy intake was associated with a higher energy intake. To assess the robustness of the associations, we adjusted for age, sex and parental educational level and subsequently repeated these analyses without the weighing factors. To correct for potential underreporting we additionally adjusted for BMR. Analyses were performed using SAS 9.2. A *p*-value of 0.05 was considered significant.

## Results

### Baseline characteristics

For children aged 7–13 years height, weight and BMI (categories) did not differ over dairy tertiles (Table 1). For adolescents, firstly higher dairy consumption was associated with higher height and weight, although no differences were seen in BMI . Secondly, higher dairy consumption was associated with less frequent achievement of the physical activity norm and less smoking.

**Table 1:** Baseline characteristics of the study population (DNFCS 2007–2010)

Age	7–13 years				14–18 years			
	Non-dairy consumers	Tertile 3	mean	P for trend	Non-dairy consumers	Tertile 3	mean	P for trend
N	17	330	1007		21	228	706	
Males (N/%)	9 (52.9 %)	184 (55.7 %)	504 (50.1 %)		13 (61.9 %)	141 (61.8 %)	352 (49.9 %)	
Height (cm) M/F	139.9(8.7)/141.5(16.5)	147.8(14.4)/147.0(14.0)	146.1(14.1)/146.3(13.8)	0.09	173.9(8.1)/168.8(5.0)	180.1(8.5)/170.6(6.6)	178.9(9.1)/169.1(6.5)	<0.0001
Weight (kg) M/F	31.3(8.0)/36.1(10.6)	38.7(12.0)/39.6(11.7)	38.0(11.9)/39.2(12.3)	0.47	64.9(10.9)/74.3(20.7)	67.7(10.9)/61.0(9.1)	67.4(12.5)/60.9(10.8)	0.02
BMI (kg/m^2^) M/F	15.9(2.8)/17.8(3.6)	17.3(2.8)/18.0(2.8)	17.4(2.8)/17.9(3.1)	0.57	21.5(3.7)/26.0(6.6)	20.9(3.0)/20.9(2.8)	21.0(3.1)/21.3(3.4)	0.37
EI/BMR	1.58(0.20)	1.79(0.24)	1.69 (0.23)	<0.0001	1.13 (0.26)	1.57(0.27)	1.46(0.27)	<0.0001
BMI category				0.75				0.45
*Seriously underweight*	3 (17.6 %)	5 (1.5 %)	28 (2.8)		0 (0.0 %)	3 (1.3 %)	10(1.4 %)	
*Underweight*	3 (17.6 %)	19 (5.8 %)	70(7.0 %)		1 (4.8 %)	12 (5.3 %)	48(6.8 %)	
*Normal weight*	8 (47.1 %)	246 (74.5 %)	715(71.2 %)		12 (57.1 %)	183 (80.3 %)	533(75.5 %)	
*Overweight*	3 (17.6 %)	52 (15.8 %)	160(15.9 %)		6 (28.6 %)	25 (11.0 %)	95(13.5 %)	
*Obese*	0 (0.0 %)	8 (2.4 %)	33(3.3 %)		2 (9.5 %)	5 (2.2 %)	20(2.8 %)	
Size of household				0.17				0.68
*1*	0 (0.0 %)	0 (0.0 %)	0(0.0 %)		1 (4.8 %)	4 (1.8 %)	11(1.6 %)	
*2 and 3*	1 (5.9 %)	76 (23.0 %)	244(24.8 %)		5 (23.8 %)	50 (21.9 %)	169(24.3 %)	
*4*	12 (70.6 %)	146 (44.2 %)	446(45.4 %)		11 (52.4 %)	102 (44.7 %)	325(46.8 %)	
*5+*	4 (23.5 %)	108 (32.7 %)	292(29.7 %)		4 (19.0 %)	72 (31.6 %)	190(27.3 %)	
Education level^a^				0.22				0.29
*Low*	3 (17.6 %)	48 (17.4 %)	127(12.6 %)		3 (14.3 %)	102 (44.7 %)	297(42.1 %)	
*Moderate*	0 (0.0 %)	13 (4.7 %)	52(5.2 %)		1 (7.1 %)	18 (7.9 %)	54(7.7 %)	
*High*	1 (5.9 %)	29 (10.5 %)	97(9.6 %)		1 (7.1 %)	8 (3.5 %)	18(2.5 %)	
Student/schoolgoing	4 (23.5 %)	88(31.8 %)	271(26.9 %)		19 (90.5 %)	213 (93.4 %)	644(91.2 %)	
Native country^b^	17(100 %)	324(98.2 %)	989(98.2 %)	0.023	21 (100 %)	223 (97.8 %)	688(97.5 %)	0.94
Physical activitity false (N/%)^c^	3 (17.6 %)	69 (24.9 %)	205(20.4 %)	0.43	15 (71.4 %)	144 (63.2 %)	497(70.4 %)	0.01
Smoking habits (yes/former/never)	0//0/4	0/1/89	0/2/275	0.37	3/0/18	16/8/204	66/28/612	0.04
Alcohol consumption yes(N/%)	0 (0.0 %)	1 (0.4 %)	9 (3.3 %)	0.24	6 (0.8 %)	112 (49.1 %)	335(47.5 %)	0.20

^a^Education level of parents was divided into low (intermediate general education); moderate (higher vocational education) and high (university)

^b^Native country: percentage of people from Dutch origin

^c^Physical Activity: indication whether the participant meets the physical activity guideline (false/true)

Education level, Physical Activity, Smoking habits and alcohol consumption: 730 missings

### Children age 7–13 years

Among children aged 7–13 years, a higher dairy consumption (693 g ±34.4 in third tertile; p-for-trend < 0.0001) was significantly associated with higher consumption of vegetables (25.0 g ±14.6; *p* = 0.001), fruits (54.8 g ±22.3; *p* < 0.0001), cereals (18.5 g ±20.7; *p* = 0.01) and fats (3.0 g ±3.7;*p* = 0.01) and lower consumption of non-alcoholic beverages (−281 g ±101; *p* < 0.0001) (Table 2): in particular soft drinks (−159 g ±28.2; *p* < 0.0001), fruit juices (−40.5 g ±14.8; *p* = 0.01) and coffee or tea (−15.5 g ±11.0; *p* = 0.01) than non-dairy consumers (data not presented). This higher dairy consumption was associated with significantly higher intakes of energy (392 kcal ±122; *p* < 0.0001), protein, fat, fibre, calcium, folate, iodine, potassium, magnesium, phosphorus, selenium, zinc, retinol activity equivalents and vitamins B1, B2, B6 and B12 compared to non-dairy consumers (Table 3).

**Table 2:** Per tertile dairy, milk and cheese consumption the intakes of food groups in gram for children aged 7 to 13 years

	Non consumers		Tertile1		Tertile2		Tertile3			Overall		
Per tertile dairy	Estimate	St error	Estimate	St error	Estimate	St error	Estimate	St error	*p*-value	Estimate	St error	*p*-value
N	17			330		330		330			1007	
Potatoes	87.9	18.1	−10.4	18.5	−5.7	18.5	−5.2	18.5	0.78	0.007	0.008	0.40
Vegetables	50.5	14.3	12.1	14.6	21.3	14.6	25.0	14.6	0.09	0.02	0.006	0.001
Fruits	46.5	21.8	31.0	22.3	46.3	22.3	54.8	22.3	0.01	0.04	0.01	<.0001
Dairy products	0.0	33.6	154.2	34.3	385.5	34.3	693.2	34.4	<.0001	0.81	0.02	<.0001
Cereals	170	20.2	2.8	20.7	4.1	20.6	18.5	20.7	0.37	0.02	0.009	0.01
Meats	78.9	14.2	6.7	14.5	9.6	14.5	2.3	14.5	0.87	−0.005	0.006	0.38
Fish	2.4	5.9	1.6	6.0	7.9	6.0	4.2	6.0	0.48	0.004	0.003	0.09
Eggs	1.2	4.0	6.2	4.1	6.6	4.1	7.9	4.1	0.06	0.003	0.002	0.09
Fats	18.8	3.7	0.3	3.7	3.4	3.7	3.0	3.7	0.42	0.004	0.002	0.01
Sugar_confectionary	85.9	14.7	−5.5	15.0	−9.2	15.0	−4.0	15.0	0.79	0.001	0.007	0.82
Cakes	51.4	13.0	4.4	13.3	7.4	13.3	5.6	13.3	0.67	0.002	0.006	0.68
Non_alcoholic_beverage	1111	99.1	−1.6	101	−143	101	−281	101	0.01	−0.42	0.04	<.0001
Alcoholic_beverage	0.0	3.7	1.3	3.8	0.4	3.8	1.8	3.8	0.63	0.001	0.002	0.63
Per tertile milk	Non consumers		Tertile1		Tertile2		Tertile3			Overall		
N	335			221		227		224			1007	
Potatoes	80.3	3.9	3.7	6.1	−1.7	6.0	0.4	6.1	0.94	−0.002	0.01	0.83
Vegetables	62.1	3.0	8.8	4.8	11.7	4.8	13.3	4.8	0.01	0.02	0.008	0.01
Fruits	85.6	4.7	−9.2	7.3	6.0	7.3	21.8	7.3	0.003	0.04	0.01	0.001
Dairy products	267	11.5	43.3	18.0	163	18.0	396	18.1	<.0001	0.68	0.03	<.0001
Cereals	172	4.3	−0.8	6.7	7.4	6.7	17.5	6.8	0.01	0.03	0.01	0.005
Meats	89.6	3.0	−6.0	4.7	−7.4	4.7	−6.7	4.7	0.16	−0.01	0.008	0.15
Fish	5.8	1.3	1.8	2.0	1.4	2.0	1.8	2.0	0.37	0.003	0.003	0.44
Eggs	7.7	0.9	0.2	1.3	−0.9	1.3	2.1	1.3	0.13	0.003	0.002	0.25
Fats	20.0	0.8	1.1	1.2	1.5	1.2	2.0	1.2	0.11	0.003	0.002	0.11
Sugar_confectionary	85.0	3.1	−11.1	4.9	−4.4	4.9	−7.9	4.9	0.11	−0.009	0.008	0.28
Cakes	57.3	2.8	−1.2	4.3	5.2	4.3	−4.8	4.3	0.27	−0.004	0.007	0.57
Non_alcoholic_beverage	1046	21.6	−18.3	33.8	−105	33.6	−206	33.9	<.0001	−0.37	0.06	<.0001
Alcoholic_beverage	0.4	0.8	1.4	1.2	2.2	1.2	−0.3	1.2	0.82	0.0000	0.002	1.00
Per tertile cheese	Non consumers		Tertile1		Tertile2		Tertile3			Overall		
N	308			233		233		233			1007	
Potatoes	92.0	3.9	−7.1	6.0	−15.6	6.0	−26.0	6.0	<.0001	−0.04	0.01	<.0001
Vegetables	63.2	3.1	5.5	4.8	9.4	4.8	13.0	4.8	0.01	0.02	0.008	0.005
Fruits	80.6	4.8	13.0	7.4	15.3	7.3	11.5	7.4	0.12	0.02	0.01	0.13
Dairy products	395	14.5	−22.6	22.1	−24.6	22.1	75.6	22.2	0.001	0.12	0.04	0.001
Cereals	154	4.2	12.5	6.4	25.5	6.4	66.7	6.5	0.0001	0.11	0.01	<.0001
Meats	89.1	3.1	−1.6	4.7	−8.0	4.7	−7.8	4.8	0.10	−0.02	0.008	0.049
Fish	8.9	1.3	−3.1	2.0	−3.1	2.0	−2.6	2.0	0.19	−0.004	0.003	0.24
Eggs	9.0	0.9	−2.2	1.3	−2.1	1.3	−0.7	1.4	0.80	0.000	0.002	0.88
Fats	21.3	0.8	−1.0	1.2	−0.7	1.2	0.6	1.2	0.63	0.001	0.002	0.58
Sugar_confectionary	87.9	3.2	−14.0	4.9	−11.0	4.9	−10.2	4.9	0.04	−0.01	0.008	0.09
Cakes	61.4	2.8	−6.0	4.3	−2.0	4.3	−10.4	4.4	0.02	−0.01	0.007	0.053
Non_alcoholic_beverage	933	22.5	30.1	34.5	44.0	34.4	99.3	34.6	0.004	0.16	0.06	0.005
Alcoholic_beverage	1.2	0.8	−0.9	1.2	−1.1	1.2	1.4	1.2	0.25	0.002	0.002	0.35

A *p*-value of 0.05 was considered significant

Tertile 1,2 and 3 represent respectively the lowest, medium and highest consumers of dairy, milk and cheese. P for trend is the p for trend over non-consumers and all three tertiles

**Table 3:** Total nutrient intake over tertiles dairy consumption in children aged 7–13 years

	Non-dairy consumers		Tertile 1		Tertile 2		Tertile 3			Overall			
Per tertile dairy	Estimate	St. error	Estimate	St. error	Estimate	St. error	Estimate	St. error	*p*-value	Estimate	St. error	p for trend	p for trend energy corrected
N	17		330		330		330			1007		1007	1007
Consumed quantity(g)	1836	125	150	128	270	128	465	128	0.0003	0.48	0.06	<.0001	<.0001
Energy (kcal)	1852	120	128	122	210	122	392	122	0.001	0.40	0.05	<.0001	<.0001
Total protein(g)	48.9	4.6	8.2	4.7	17.7	4.7	27.8	4.7	<.0001	0.03	0.002	<.0001	<.0001
Vegetable protein(g)	27.6	1.96	−2.5	2.0	−1.8	2.0	−0.54	2.0	0.79	0.003	0.001	0.003	0.03
Animal protein(g)	21.4	3.9	10.6	4.0	19.3	4.0	28.2	4.0	<.0001	0.03	0.002	<.0001	0.29
Total fat(g)	66.7	6.5	9.9	6.7	11.0	6.7	15.3	6.7	0.02	0.009	0.003	0.002	<.0001
Saturated fatty acids(g)	19.7	2.6	8.2	2.6	9.4	2.6	12.7	2.6	<.0001	0.007	0.001	<.0001	0.37
Mono-unsaturated fatty acids cis(g)	26.5	2.6	1.54	2.6	1.05	2.6	1.72	2.6	0.51	0.0004	0.001	0.75	<.0001
Poly-unsaturated fatty acids(g)	15.9	1.59	−1.27	1.62	−1.21	1.62	−1.31	1.63	0.42	0.0002	0.001	0.80	<.0001
Trans fatty acids(g)	0.56	0.19	0.00	0.19	0.67	0.19	0.85	0.19	<.0001	0.0004	0.000	<.0001	0.048
N-3 fish fatty acids (EPA + DHA.mg)	28.7	53.7	21.4	54.9	72.6	54.9	41.5	54.9	0.45	0.03	0.02	0.15	0.31
Total carbohydrates(g)	256	16.4	1.23	16.7	9.3	16.7	33.7	16.7	0.04	0.05	0.007	<.0001	0.80
Mono- and disaccharides(g)	135	11.7	−1.25	12.0	7.0	12.0	26.2	12.0	0.03	0.04	0.005	<.0001	0.00
Polysaccharides(g)	120	8.5	2.6	8.70	2.4	8.7	7.6	8.7	0.38	0.008	0.004	0.047	<.0001
Fibre(g)	15.3	1.23	−0.23	1.25	1.06	1.25	2.4	1.26	0.053	0.004	0.001	<.0001	0.00
Alcohol(g)	0.00	0.08	0.04	0.08	0.02	0.08	0.05	0.08	0.56	0.000	0.000	0.74	0.77
Calcium(mg)	387	67.7	231	69.2	485	69.2	887	69.2	<.0001	0.99	0.03	<.0001	<.0001
Copper(mg)	0.87	0.07	0.02	0.07	0.08	0.07	0.14	0.07	0.04	0.000	0.0003	<.0001	0.47
Iron(mg)	8.2	0.64	−0.31	0.66	0.21	0.66	0.90	0.66	0.17	0.002	0.0003	<.0001	0.11
Folate equivalents(μg)	135	19.8	27.8	20.3	47.9	20.3	77.7	20.3	0.0001	0.08	0.009	<.0001	<.0001
Iodine(μg)	107	12.9	21.2	13.2	43.5	13.2	64.1	13.2	<.0001	0.07	0.006	<.0001	<.0001
Potassium(mg)	2075	168	74.5	171	460	171	942	171	<.0001	1.29	0.07	<.0001	<.0001
Magnesium(mg)	225	16.9	−9.3	17.2	21.1	17.2	68.5	17.3	<.0001	0.11	0.008	<.0001	0.0004
Sodium(mg)	1802	184	405	188	494	188	636	189	0.0008	0.38	0.08	<.0001	0.61
Phosphorus(mg)	788	73.2	204	74.8	443	74.8	751	74.8	<.0001	0.83	0.03	<.0001	<.0001
Selenium(μg)	25.2	3.3	6.5	3.4	9.9	3.4	11.8	3.4	0.0004	0.009	0.001	<.0001	0.02
Zinc(mg)	5.8	0.71	1.53	0.72	2.5	0.72	3.9	0.72	<.0001	0.004	0.0003	<.0001	<.0001
Retinol activity equivalents(μg)	445	131	89.1	134	216	134	283	134	0.04	0.30	0.06	<.0001	0.004
Vitamin B1(mg)	0.89	0.12	−0.02	0.13	0.09	0.13	0.17	0.13	0.17	0.000	0.0001	<.0001	0.02
Vitamin B2(mg)	0.71	0.12	0.26	0.12	0.66	0.12	1.22	0.12	<.0001	0.001	0.0001	<.0001	<.0001
Vitamin B6(mg)	1.29	0.21	0.16	0.22	0.33	0.22	0.50	0.22	0.02	0.001	0.0001	<.0001	0.002
Vitamin B12(μg)	1.27	0.44	1.15	0.45	2.3	0.45	3.3	0.45	<.0001	0.003	0.0002	<.0001	<.0001
Vitamin C(mg)	98	12.5	−14.6	12.7	−14.4	12.7	−18.2	12.8	0.16	−0.006	0.006	0.24	0.001
Vitamin D(μg)	1.83	0.38	0.37	0.39	0.80	0.38	0.88	0.39	0.02	0.001	0.0002	<.0001	0.08
Vitamin E(mg)	11.9	1.26	−0.94	1.29	−0.54	1.29	−0.35	1.29	0.79	0.001	0.001	0.15	0.01

A *p*-value of 0.05 was considered significant

Tertile 1,2 and 3 represent respectively the lowest, medium and highest dairy consumers

P for trend is the p for trend over non-consumers and all three tertiles

Higher dairy consumption was associated with significantly lower nutrient intake from non-dairy sources such as energy (−229 kcal ±126), (vegetable) protein (−0.81 g ±2.0), and higher intakes of fibre (1.18 g ±1.2), iron (0.28 mg ±0.64), folate (31.6 μg ±19.6), iodine (23.0 μg ±12.7), retinol equivalents (109 μg ±132), and vitamin D (0.73 μg ±0.38)(Data not shown). Only 17 children (1.7 %) did not consume any dairy on both recall days.

For milk consumption, we observed similar associations with higher consumption of vegetables and fruits (Table 2) and with lower consumption of soft drinks (−159 g ±28.2), fruit juice (−40.5 g ±14.8) and less tea or coffee (−15.5 g ±11.0) as found for total dairy consumption. As milk contained only milk and buttermilk, higher milk consumption was also associated with lower intakes of other milk beverages (−13.7 g ±7.7) and especially less yoghurt (−68.5 g ±14.6). Comparable to higher dairy intake, higher milk intake was significantly associated with the same nutrients as for dairy consumption (Additional file 1). 335 children (33.3 %) did not drink any milk on both recall days.

For cheese consumption we observed associations similar to dairy with a higher consumption of vegetables, cereals and non-alcoholic beverages, but lower consumption of potatoes (−26.0 g ±6.0) and meat (−7.8 g ±4.8) than non-cheese consumers (Table 2). A higher cheese consumption was associated with lower consumption of chocolate spread (data not shown). Higher cheese consumption was significantly associated with higher intakes of energy, protein, SFAs, TFAs, calcium, sodium and several vitamins and minerals such as potassium, zinc and vitamin A (Additional file 2). 308 children (30.6 %) did not consume any cheese on both recall days.

### Adolescents age 14–18 years

In adolescents, similar results were found as for children except for the significant associations of dairy and milk consumption with higher fruit and vegetables intakes. Higher dairy consumption was associated with higher consumption of cereals (50.3 g ±22.8; p-for-trend = 0.01), and lower intakes of non-alcoholic beverages (−299 g ±116; *p* < 0.0001) (Table 4): in particular soft drinks (−96.5 g ±94.2; *p* = 0.0001) and coffee or tea (−15.3 g ±54.4; *p* < 0.0001) than non-dairy consumers (Data not presented). High dairy consumption was associated with significantly higher intakes of energy, animal protein, fat, fibre, calcium and other dairy predominant nutrients. Higher dairy consumption was associated with significantly higher intakes of nutrients from non-dairy products such as vegetable protein (5.3 g ±2.3), fibre (3.8 g ±1.42), iron (1.01 mg ±0.67), folate (58.1 μg ±20.0), potassium (269 mg ±177), magnesium (22.5 mg ±19.9) and vitamin D (0.94 μg ±0.37) and lower energy intakes (−309 kcal ±166) from non-dairy products. (data not shown) 3 % (*N* = 21) adolescents did not consume any dairy on both recall days.

**Table 4:** Per tertile dairy. milk and cheese consumption the intakes of food groups in gram for children aged 14 to 18 years

	Non consumers		Tertile1		Tertile2		Tertile3		Overall		
Per tertile dairy	Estimate	St error	Estimate	St error	Estimate	St error	Estimate	St error	Estimate	St error	*p*-value
N	21		228		229		228			706	
Potatoes	96.2	17.3	−7.1	18.2	6.8	18.3	−0.63	18.2	0.01	0.02	0.46
Vegetables	83.5	13.3	8.2	13.9	3.6	14.0	12.7	13.9	0.01	0.01	0.36
Fruits	35.9	19.9	50.9	20.9	45.0	20.9	55.6	20.9	0.02	0.02	0.19
Dairy products	0.00	29.7	127.1	31.2	354	31.3	707	31.1	1.26	0.03	<.0001
Cereals	192	21.7	27.4	22.8	21.1	22.8	50.3	22.8	0.06	0.02	0.007
Meats	116	13.1	−13.7	13.8	−10.2	13.8	−16.1	13.7	−0.009	0.01	0.47
Fish	2.9	4.6	4.0	4.8	2.4	4.8	6.0	4.8	0.005	0.004	0.23
Eggs	5.4	3.2	3.6	3.4	1.30	3.4	2.8	3.4	−0.001	0.003	0.81
Fats	16.7	3.6	7.6	3.8	5.9	3.8	8.1	3.8	0.003	0.003	0.35
Sugar_confectionary	48.7	10.8	11.5	11.3	7.4	11.3	15.8	11.3	0.01	0.01	0.23
Cakes	31.5	10.7	19.6	11.3	23.4	11.3	25.8	11.3	0.02	0.01	0.06
Non_alcoholic_beverage	1504	111	−30.4	116	−155	117	−299	116	−0.58	0.10	<.0001
Alcoholic_beverage	221	83.8	−166.5	88.0	−124	88.2	−120	87.9	0.05	0.08	0.53
Per tertile milk	Non consumers		Tertile1		Tertile2		Tertile3		Overall		
N	231		160		157		158			706	
Potatoes	89.2	5.5	4.9	8.7	23.1	8.8	2.3	8.6	0.02	0.02	0.40
Vegetables	83.0	4.2	16.7	6.7	13.8	6.7	7.9	6.6	0.02	0.02	0.33
Fruits	80.8	6.4	11.6	10.1	−1.82	10.2	8.1	9.9	0.009	0.02	0.71
Dairy products	205	14.0	91.4	22.2	213	22.3	500	21.8	1.20	0.05	<.0001
Cereals	226	7.0	−14.1	11.0	−10.6	11.1	14.9	10.8	0.04	0.03	0.16
Meats	105	4.2	−2.5	6.6	0.98	6.7	−7.6	6.5	−0.01	0.02	0.34
Fish	5.0	1.47	0.89	2.3	3.1	2.3	4.8	2.3	0.01	0.01	0.02
Eggs	8.4	1.03	0.54	1.63	−0.91	1.64	−1.89	1.60	−0.005	0.004	0.17
Fats	23.8	1.16	−1.49	1.83	0.58	1.85	0.16	1.81	0.002	0.004	0.67
Sugar_confectionary	57.6	3.5	4.4	5.4	2.2	5.5	4.1	5.4	0.008	0.01	0.55
Cakes	49.9	3.4	6.6	5.4	2.2	5.5	8.1	5.4	0.02	0.01	0.23
Non_alcoholic_beverage	1452	35.5	−52.2	56.1	−153	56.5	−267	55.3	−0.67	0.13	<.0001
Alcoholic_beverage	91.6	26.9	−23.1	42.4	2.1	42.7	7.7	41.8	0.04	0.10	0.72
Per tertile cheese	Non consumers		Tertile1		Tertile2		Tertile3		Overall		
N	192		165		179		170			706	
Potatoes	101	6.1	−3.9	9.0	5.8	8.8	−22.2	8.9	−0.04	0.02	0.03
Vegetables	84.2	4.7	0.99	6.9	10.0	6.7	18.3	6.8	0.04	0.01	0.003
Fruits	65.8	7.0	28.5	10.3	15.2	10.0	35.0	10.2	0.06	0.02	0.01
Dairy products	355	20.8	22.0	30.6	34.6	29.8	74.3	30.1	0.16	0.07	0.01
Cereals	205	7.5	−3.2	11.0	10.9	10.7	69.7	10.8	0.16	0.02	<.0001
Meats	109	4.6	−6.4	6.8	−3.9	6.6	−13.4	6.7	−0.03	0.01	0.08
Fish	9.1	1.63	−2.6	2.4	−1.87	2.3	−4.4	2.4	−0.01	0.005	0.11
Eggs	6.9	1.14	2.3	1.68	1.28	1.64	0.31	1.66	−0.001	0.004	0.86
Fats	21.9	1.28	−1.44	1.88	4.4	1.83	3.6	1.85	0.01	0.004	0.004
Sugar_confectionary	57.3	3.8	7.7	5.6	4.2	5.5	−1.00	5.5	−0.01	0.01	0.59
Cakes	57.3	3.8	−2.9	5.6	−7.9	5.5	−3.8	5.5	−0.01	0.01	0.39
Non_alcoholic_beverage	1291	39.8	−37.7	58.5	113	56.9	140	57.6	0.40	0.12	0.001
Alcoholic_beverage	63.9	29.7	23.9	43.6	−9.1	42.5	88.7	43.0	0.16	0.09	0.08

A *p*-value of 0.05 was considered significant

Tertile 1,2 and 3 represent respectively the lowest, medium and highest consumers of dairy, milk and cheese. P for trend is the p for trend over non-consumers and all three tertiles

Higher milk consumption was associated with lower consumption of non-alcoholic beverages (Table 4), soft drinks (−159 g ±44.8) and coffee or tea (−93.2 g ±25.7) (Data not presented) and higher intakes of fish (4.8 g ±2.3) than non-milk consumers. Comparable to children, higher milk consumption in adolescents was significantly associated with higher intakes of energy and other milk nutrients (Additional file 3). 32.7 % (*N* = 231) of the adolescents did not drink any milk on both recall days.

Comparable to children, higher cheese consumption by adolescents was associated with higher consumption of vegetables, cereals, and non-alcoholic beverages and lower intakes of potatoes (Table 4) and chocolate spread (5 g) (Data not presented). In contrast to children, higher cheese consumption was associated with higher fruit and fat consumption, but not with lower meat consumption. Higher cheese consumption was again associated with higher intakes of energy, protein, SFA, TFA, calcium, sodium and other dairy nutrients (Additional file 4). 27.2 % (*N* = 192) of the adolescents did not consume any cheese on both recall days.

### Adjustments

Adjustment for energy intake did not alter our results, although higher dairy consumption was associated lower intakes of vitamin C and vitamin E than non-dairy consumers and the associations with SFA and sodium lost significance in children (Table 3) and the association between higher dairy consumption with TFA and sodium lost significance in adolescents (Table 5). Additional correction for age, sex and education of parents did not alter the results in children with the exception of milk intake, which was no longer associated with fruit and cereals; and cheese intake was no longer associated with potatoes and meats.

**Table 5:** Total nutrient intake over tertiles dairy consumption in children aged 14–18 years

	Non-dairy consumers		Tertile 1		Tertile 2		Tertile 3			Overall			
Per tertile dairy	Estimate	St. error	Estimate	St. error	Estimate	St. error	Estimate	St. Error	*p*-value	Estimate	St. error	p for trend	p for trend energy corrected
N	21		228		229		228			706			
Consumed quantity (g)	2491	161	3.3	169	139	170	398	169	0.02	0.83	0.15	<.0001	0.04
Energy (kcal)	1879	150	335	157	441	158	726	157	<.0001	0.92	0.14	<.0001	xx
Total protein(g)	60.6	4.7	8.8	5.0	16.0	5.0	30.9	5.0	<.0001	0.05	0.004	<.0001	<.0001
Vegetable protein(g)	28.2	2.2	2.0	2.3	2.3	2.3	5.5	2.3	0.02	0.008	0.002	0.0002	0.08
Animal protein(g)	30.9	3.7	8.1	3.9	15.1	3.9	26.7	3.9	<.0001	0.04	0.003	<.0001	<.0001
Total fat(g)	71.0	7.3	13.9	7.7	17.2	7.7	25.0	7.6	0.001	0.03	0.007	<.0001	0.00
Saturated fatty acids(g)	22.0	2.7	8.7	2.8	10.0	2.8	15.4	2.8	<.0001	0.02	0.003	<.0001	0.03
Mono-unsaturated fatty acids cis(g)	27.3	2.9	3.2	3.0	4.2	3.0	6.0	3.0	0.047	0.007	0.003	0.01	<.0001
Poly-unsaturated fatty acids(g)	15.5	1.78	1.27	1.87	1.86	1.88	1.89	1.87	0.31	0.002	0.002	0.31	<.0001
Trans fatty acids(g)	1.11	0.16	0.17	0.17	0.25	0.17	0.41	0.17	0.02	0.0005	0.15	0.0004	0.69
N-3 fish fatty acids (EPA + DHA.mg)	41.6	49.9	55.0	52.4	21.9	52.5	60.1	52.3	0.25	0.02	0.05	0.60	0.64
Total carbohydrates(g)	223	18.2	53.4	19.1	62.6	19.2	99.2	19.1	<.0001	0.11	0.02	<.0001	0.13
Mono- and disaccharides(g)	94.5	11.6	41.9	12.2	45.2	12.2	71.9	12.2	<.0001	0.08	0.01	<.0001	0.001
Polysaccharides(g)	129	10.1	11.5	10.6	17.5	10.6	27.4	10.6	0.01	0.04	0.009	0.0001	0.002
Fibre(g)	16.4	1.38	1.83	1.45	2.6	1.45	5.1	1.45	0.0004	0.007	0.001	<.0001	0.08
Alcohol(g)	9.7	3.9	−6.2	4.12	−5.1	4.1	−4.7	4.1	0.26	0.001	0.004	0.71	0.02
Calcium(mg)	374	67.1	296	70.6	553	70.7	1026	70.4	<.0001	1.63	0.06	<.0001	<.0001
Copper(mg)	0.99	0.08	0.07	0.08	0.12	0.08	0.24	0.08	0.00	0.000	0.07	<.0001	0.79
Iron(mg)	8.9	0.65	0.23	0.68	0.62	0.69	1.58	0.68	0.02	0.003	0.61	<.0001	0.64
Folate equivalents(μg)	160	19.5	43.9	20.5	55.9	20.5	108	20.5	<.0001	0.15	0.02	<.0001	<.0001
Iodine(μg)	149	12.6	2.4	13.3	16.9	13.3	51.6	13.3	0.0001	0.10	0.01	<.0001	<.0001
Potassium(mg)	2217	179	296	188	697	189	1336	188	<.0001	2.3	0.17	<.0001	<.0001
Magnesium(mg)	241	19.9	18.5	21.0	55.4	21.0	113	20.9	<.0001	0.21	0.02	<.0001	<.0001
Sodium(mg)	2312	187	264	196	348	197	607	196	0.002	0.80	0.18	<.0001	0.63
Phosphorus(mg)	981	86.9	212	91.4	438	91.5	836	91.2	<.0001	1.38	0.08	<.0001	<.0001
Selenium(μg)	36.9	3.1	2.3	3.3	3.4	3.3	7.9	3.3	0.02	0.01	0.003	<.0001	0.45
Zinc(mg)	8.5	0.67	0.25	0.70	1.03	0.71	3.0	0.70	<.0001	0.006	0.63	<.0001	<.0001
Retinol activity equivalents(μg)	471	161	189	169	209	170	289	169	0.09	0.27	0.15	0.08	0.64
Vitamin B1(mg)	0.94	0.11	0.10	0.12	0.14	0.12	0.29	0.12	0.02	0.0004	0.11	<.0001	0.05
Vitamin B2(mg)	0.83	0.13	0.26	0.14	0.61	0.14	1.33	0.14	<.0001	0.002	0.13	<.0001	<.0001
Vitamin B6(mg)	1.77	0.22	0.07	0.24	0.19	0.24	0.47	0.24	0.05	0.001	0.21	<.0001	0.12
Vitamin B12(μg)	2.1	0.45	0.96	0.47	1.56	0.47	3.0	0.47	<.0001	0.005	0.43	<.0001	<.0001
Vitamin C(mg)	75.2	12.5	19.5	13.1	17.8	13.2	28.2	13.1	0.03	0.02	0.01	0.04	0.28
Vitamin D(μg)	1.97	0.36	0.72	0.38	0.79	0.38	1.11	0.38	0.003	0.001	0.34	0.002	0.59
Vitamin E(mg)	11.5	1.52	1.26	1.60	1.83	1.60	2.2	1.59	0.17	0.002	0.001	0.10	0.01

A *p*-value of 0.05 was considered significant

Tertile 1,2 and 3 represent respectively the lowest, medium and highest dairy consumers

P for trend is the p for trend over non-consumers and all three tertiles

In adolescents, only in the dairy category cereals lost significance, and the association with meat became significant for dairy, milk and cheese (data not shown).

Additional correction for BMR did not alter any of the results. Analyses without the weighting factors to adjust demographic properties, season and the day combination of both consumption days did not change the results.

## Discussion

In this Dutch sample of children and adolescents, higher dairy consumption was associated with higher intakes of cereals and lower consumption of non-alcoholic beverages, especially soft drinks. Among children higher dairy consumption was associated with higher intakes of vegetables and fruits, but less fruit juices. Higher cheese intakes were associated with higher consumption of vegetables and non-alcoholic beverages and lower consumption of potatoes in both age categories and with lower meat consumption in children. These associations were also reflected in the nutrient intakes such as protein, fat, MUFA, calcium, vitamin B2, and vitamin B12.

These findings confirm that competing foods such as soda may replace dairy products as mentioned by Nicklas [17] and that higher consumption of dairy foods might be a marker for healthier eating habits [9]. This knowledge might be helpful for recommendations to ensure adequate nutrient intakes in children and adolescents.

In the Netherlands, children aged 4 to 8 years are recommended to consume 400 ml of milk and 10 gram cheese, while for adolescents 600 ml of milk and 20 gram cheese is recommended, both preferably low fat [7]. Our results show that 30 % of the children and adolescents did not consume milk and over 27 % did not consume cheese on the two days. Therefore, a substantial proportion of children and especially adolescents may fail to meet these recommendations. The consumption of dairy products was highest in children and decreased with age [8]. In other developed countries the proportion of children and adolescents meeting dairy product intake recommendation also tends to decrease with age [2, 4, 5], although Green et al. found no difference in milk-based dairy consumption between middle-childhood and adolescence [6].

The Dutch National Food Consumption Survey 2007–2010 [8] showed that the consumption of fruits, vegetables, fish and fibre is insufficient in children and adolescents. We found that higher dairy consumption in children and higher cheese consumption in adolescents was associated with higher intakes of fruits and vegetables. This is in line with a previous study in Australian children (aged 8 to 10 years) which also found that dairy intake was associated with higher intakes of bread and cereals and lower intakes of meat [9]. Although this study also observed slightly higher intakes of fruit and vegetables with higher dairy intake, these associations did not reach significance, like the adolescents in our study. Overall, our results and the study by Rangan et al. suggests that dairy may contribute to nutrient intakes by contributing nutrients from dairy itself, but also through associations with nutrient intakes from non-dairy food groups. Indeed, in our study higher dairy consumption was associated with increased nutrient intake from non-dairy sources such as fibre, protein, iodine and vitamin D. Moreover, higher dairy and milk consumption was associated with lower consumption of soft drinks and coffee or tea, suggesting that dairy products were mainly replaced by these foods. This is in line with other studies reporting that lower intakes of milk were indeed associated with higher intakes of softdrinks in adolescents [9, 18].

The Dutch National Food Consumption Survey 2007–2010 [8] showed potential inadequacies for vitamin A, C and E, potassium, magnesium and zinc for both children and adolescents. Especially adolescents do not seem to meet the age-specific higher calcium requirements. In contrast, the proportion of SFAs in the diet and sodium intake are too high in both children and adolescents. As a higher dairy intake was associated with higher intakes of vitamin A, calcium, potassium, magnesium and zinc, dairy could thus contribute to the adequate intakes of these nutrients. In addition, dairy intakes were associated with higher intakes of fruits, vegetables, and cereals and could indirectly contribute to higher intakes of vitamin B1 and fibre in children. For vitamin C, we observed opposite results for adolescents and children, with higher dairy intakes, intake of vitamin C was higher among adolescents and lower among children. Consistent with our results, Rangan et al. also showed higher intakes nutrients from dairy, like calcium, potassium, magnesium, zinc, vitamin A and vitamin B2 [9]. However, they did not detect significant associations for vitamin B1, C and fibre. This could be due to the fact that they found no significant associations of dairy consumption with fruit and vegetable intake, while we did in children. Another difference is the adjustment for age, sex and education that was performed by Ragnan, while we only adjusted for energy intake. However, we adjusted for age, sex and parental education in sensitivity analyses and this did not fully explain our results.

On the other hand, higher dairy consumption was also associated with higher energy intake and higher SFAs and TFAs in the diet and with higher sodium intake for cheese consumption. Approximately 65 % of milk fatty acids are saturated [19]. Therefore, dairy products may contribute to the excess intake of SFA in the Dutch population. A high intake of saturated fat is associated with increased risks of cardiovascular diseases and other chronic diseases [20]. In addition, a high sodium intake is associated with an increased risk of hypertension and cardiovascular disease [21]. Despite the contribution of dairy to high intakes of SFAs, TFAs and sodium, prospective cohort studies generally reported neutral or inverse associations between dairy intake and cardiovascular disease [22–24]. This could be due to counteracting effect of other nutrients in dairy such as potassium [25] and magnesium [26] that are associated with a decreased risk of hypertension. The association of calcium with risk of cardiovascular disease is still debated [27], but the effects in the range of habitual dietary intake is likely minimal [28]. Finally, the contribution of high dairy intake to excess energy intake may contribute to adiposity. Despite this, high dairy intake was associated with lower adiposity in adolescents and no association was found in children [3]. Furthermore, dairy products showed associations with linear growth and bone health during childhood [2, 29]. In line with these results, BMI of children and adolescents did not differ according to dairy intake in our study. Since physical activity was higher with higher dairy intake, this could to some extent explain why BMI was not higher with higher dairy and energy intake. Another explanation could be underreporting of dietary intake, and thus also dairy intake, in the non-dairy consumers. We have compared potential underreporting over the dairy categories based on the ratio of energy intake to basal metabolic rate. We indeed observed that this ratio was lower among non-dairy consumers, which could indicate a higher level of underreporting in that category. However, as their physical activity level for adolescents was also lower, this may also explain the differences in ratio of energy intake to basal metabolic rate, but not for children.

Strengths of this study include the dietary assessment using a validated non-consecutive 24-h recall method [11, 12] and the adequate representation of Dutch children and adolescents. However, although two recalls are sufficient to estimate mean intake, one would ideally use more recall days to rank participants correctly from high to low intake. The use of two recall days may lead to misclassification over the dairy categories and probably underestimates the true dairy intake for the non-consumers and overestimates the true dairy intake for the highest tertile. Furthermore the percentage of absolute non-consumers is probably somewhat lower as some people who did not report dairy on one of the recall days may occasionally still consume dairy. Therefore, the percentage of children or adolescents not meeting the dairy recommendations should be interpreted with caution. A further limitation of this study is the use of self-reported height and weight [30].

## Conclusions

Higher milk and dairy consumption were associated with lower non-alcoholic beverages consumption, and higher cereal, fruit and vegetable consumption in children, which was also reflected in the nutrient intakes. These findings confirm that the consumption of milk and dairy products might be marker for healthier eating habits. This knowledge might be helpful for recommendations to ensure adequate nutrient intakes in children and adolescents.

## Additional files

Additional file 1:
**Total nutrient intake over tertiles milk consumption in children aged 7–13 years.** A *p*-value of 0.05 was considered significant. Tertile 1,2 and 3 represent respectively the lowest, medium and highest milk consumers. P for trend is the p for trend over non-consumers and all three tertiles. (DOCX 19 kb) Additional file 2:
**Total nutrient intake over tertiles cheese consumption in children aged 7–13 years.** A *p*-value of 0.05 was considered significant. Tertile 1,2 and 3 represent respectively the lowest, medium and highest cheese consumers. P for trend is the p for trend over non-consumers and all three tertiles. (DOCX 18 kb) Additional file 3:
**Total nutrient intake over tertiles milk consumption in children aged 14–18 years.** A *p*-value of 0.05 was considered significant. Tertile 1,2 and 3 represent respectively the lowest, medium and highest milk consumers. P for trend is the p for trend over non-consumers and all three tertiles. (DOCX 22 kb) Additional file 4:
**Total nutrient intake over tertiles cheese consumption in children aged 14–18 years.** A *p*-value of 0.05 was considered significant. Tertile 1,2 and 3 represent respectively the lowest, medium and highest cheese consumers. P for trend is the p for trend over non-consumers and all three tertiles. (DOCX 19 kb)

## Abbreviations

BMI: body mass index
BMR: basal metabolism rate
MUFA: mono-unsaturated fatty acids
SFA: saturated fatty acids
TFA: trans-fatty acids
