# Supplementary material for: The association of dairy intake of children and adolescents with different food and nutrient intakes in the Netherlands
Source: BMC Pediatr. 2016 Jan 9;16:2. doi: 10.1186/s12887-015-0524-3 (PMC4707007; doi:10.1186/s12887-015-0524-3)
Supplement: Additional file 1: — Total nutrient intake over tertiles milk consumption in children aged 7–13 years. A p-value of 0.05 was considered significant. Tertile 1,2 and 3 represent respectively the lowest, medium and highest milk consumers. P for trend is the p for trend over non-consumers and all three tertiles. (DOCX 19 kb) [file 12887_2015_524_MOESM1_ESM.docx]

**Additional file 1. Total nutrient intake over tertiles milk consumption in children aged 7-13 years**

|  | Non-milk consumers | | Tertile 1 | | Tertile 2 | | Tertile 3 | | | overall | | | |
| --- | --- | --- | --- | --- | --- | --- | --- | --- | --- | --- | --- | --- | --- |
| Per tertile milk | estimate | St. error | estimate | St. error | estimate | St. error | estimate | St. error | p-value | estimate | St. error | p for trend | p for trend  energy corrected |
| N | 335 |  | 221 |  | 227 |  | 224 |  |  | 1007 |  |  | 1007 |
| Consumed quantity (g) | 2062 | 27.3 | 7 | 43 | 58 | 43 | 216 | 42.9 | <.0001 | 0.37 | 0.07 | <.0001 | <.0001 |
| Energy (kcal) | 2059 | 26.1 | -17 | 41 | 45 | 41 | 110 | 41.0 | 0.01 | 0.21 | 0.07 | 0.003 | <.0001 |
| Total protein(g) | 61.8 | 1.04 | 1.19 | 1.63 | 5.4 | 1.6 | 13.9 | 1.6 | .0001 | 0.02 | 0.003 | <.0001 | <.0001 |
| Vegetable protein(g) | 25.6 | 0.42 | -0.23 | 0.66 | 1.10 | 0.65 | 1.00 | 0.66 | 0.13 | 0.002 | 0.001 | 0.04 | 0.81 |
| Animal protein(g) | 36.2 | 0.89 | 1.40 | 1.40 | 4.3 | 1.39 | 12.9 | 1.40 | <.0001 | 0.02 | 0.002 | <.0001 | <.0001 |
| Total fat(g) | 76.7 | 1.40 | 1.08 | 2.2 | 2.8 | 2.2 | 4.7 | 2.2 | 0.03 | 0.01 | 0.004 | 0.03 | 0.61 |
| Saturated fatty acids(g) | 28.3 | 0.56 | 0.19 | 0.87 | 1.91 | 0.87 | 3.9 | 0.87 | <.0001 | 0.01 | 0.001 | <.0001 | <.0001 |
| Mono-unsaturated fatty acids cis(g) | 27.9 | 0.55 | -0.11 | 0.86 | 0.08 | 0.85 | -0.08 | 0.86 | 0.92 | -0.00003 | 0.001 | 0.99 | 0.0001 |
| Poly-unsaturated fatty acids(g) | 14.4 | 0.34 | 0.91 | 0.53 | 0.37 | 0.53 | 0.09 | 0.53 | 0.87 | -0.0001 | 0.001 | 0.87 | 0.01 |
| Trans fatty acids(g) | 1.15 | 0.04 | 0.03 | 0.06 | 0.15 | 0.06 | 0.27 | 0.06 | <.0001 | 0.0005 | 0.0001 | <.0001 | 0.0003 |
| N-3 fish fatty acids (EPA+DHA.mg) | 60.9 | 11.5 | 12.7 | 18.0 | 26.8 | 17.9 | 16.1 | 18.1 | 0.37 | 0.03 | 0.03 | 0.28 | 0.36 |
| Total carbohydrates(g) | 271 | 3.6 | -7.6 | 5.6 | -0.77 | 5.6 | 2.5 | 5.6 | 0.66 | 0.01 | 0.01 | 0.42 | 0.001 |
| Mono- and disaccharides(g) | 147 | 2.6 | -6.7 | 4.0 | -1.53 | 4.0 | 1.34 | 4.0 | 0.74 | 0.005 | 0.01 | 0.47 | 0.11 |
| Polysaccharides(g) | 124 | 1.82 | -0.89 | 2.8 | 0.78 | 2.8 | 1.10 | 2.9 | 0.70 | 0.003 | 0.005 | 0.58 | 0.01 |
| Fibre(g) | 16.2 | 0.27 | -0.27 | 0.42 | 0.51 | 0.42 | 0.75 | 0.42 | 0.07 | 0.002 | 0.001 | 0.02 | 0.47 |
| Alcohol(g) | 0.03 | 0.02 | 0.003 | 0.03 | 0.004 | 0.03 | -0.01 | 0.03 | 0.57 | -0.00002 | <0.0001 | 0.61 | 0.59 |
| Calcium(mg) | 736 | 17.7 | 49.5 | 27.8 | 206 | 27.6 | 520 | 27.8 | <.0001 | 0.90 | 0.05 | <.0001 | <.0001 |
| Copper(mg) | 0.94 | 0.01 | -0.02 | 0.02 | 0.03 | 0.02 | 0.02 | 0.02 | 0.50 | 0.0001 | <0.0001 | 0.17 | 0.28 |
| Iron(mg) | 8.3 | 0.14 | -0.20 | 0.22 | 0.47 | 0.22 | 0.63 | 0.22 | 0.004 | 0.001 | 0.0004 | 0.0002 | 0.02 |
| Folate equivalents(µg) | 167 | 4.2 | 3.9 | 6.6 | 24.7 | 6.6 | 52.5 | 6.7 | <.0001 | 0.09 | 0.01 | <.0001 | <.0001 |
| Iodine(µg) | 137 | 2.8 | 0.34 | 4.4 | 16.7 | 4.4 | 36.9 | 4.4 | <.0001 | 0.07 | 0.01 | <.0001 | <.0001 |
| Potassium(mg) | 2334 | 38.4 | 76.7 | 60.2 | 268 | 59.9 | 639 | 60.3 | <.0001 | 1.11 | 0.10 | <.0001 | <.0001 |
| Magnesium(mg) | 234 | 3.8 | -1.45 | 6.0 | 21.9 | 5.9 | 56.0 | 6.0 | <.0001 | 0.10 | 0.01 | <.0001 | <.0001 |
| Sodium(mg) | 2260 | 39.6 | 14.9 | 62.1 | 15.0 | 61.8 | 168 | 62.2 | 0.01 | 0.26 | 0.10 | 0.01 | 0.48 |
| Phosphorus(mg) | 1096 | 17.7 | 45.9 | 27.8 | 178 | 27.6 | 439 | 27.8 | <.0001 | 0.76 | 0.05 | <.0001 | <.0001 |
| Selenium(µg) | 34 | 0.71 | -0.22 | 1.11 | 0.67 | 1.10 | 3.4 | 1.11 | 0.002 | 0.01 | 0.002 | 0.002 | 0.07 |
| Zinc(mg) | 7.7 | 0.15 | 0.06 | 0.24 | 0.78 | 0.24 | 2.1 | 0.24 | <.0001 | 0.004 | 0.0004 | <.0001 | <.0001 |
| Retinol activity equivalents(µg) | 584 | 28.2 | -7.8 | 44.1 | 106 | 43.9 | 141 | 44.2 | 0.002 | 0.28 | 0.07 | 0.0001 | 0.003 |
| Vitamin B1(mg) | 0.90 | 0.03 | 0.06 | 0.04 | 0.08 | 0.04 | 0.17 | 0.04 | <.0001 | 0.0003 | 0.0001 | <.0001 | 0.002 |
| Vitamin B2(mg) | 1.15 | 0.03 | 0.10 | 0.05 | 0.29 | 0.05 | 0.75 | 0.05 | <.0001 | 0.001 | 0.0001 | <.0001 | <.0001 |
| Vitamin B6(mg) | 1.59 | 0.05 | -0.01 | 0.07 | 0.04 | 0.07 | 0.06 | 0.07 | 0.39 | 0.0001 | 0.0001 | 0.31 | 0.92 |
| Vitamin B12(µg) | 2.7 | 0.10 | 0.28 | 0.15 | 0.98 | 0.15 | 2.2 | 0.15 | <.0001 | 0.004 | 0.0003 | <.0001 | <.0001 |
| Vitamin C(mg) | 86 | 2.7 | -3.6 | 4.2 | -4.8 | 4.1 | -8.5 | 4.2 | 0.04 | -0.01 | 0.01 | 0.05 | 0.004 |
| Vitamin D(µg) | 2.4 | 0.08 | 0.00 | 0.13 | 0.13 | 0.13 | 0.19 | 0.13 | 0.13 | 0.0004 | 0.0002 | 0.08 | 0.62 |
| Vitamin E(mg) | 11.1 | 0.27 | 0.18 | 0.42 | 0.67 | 0.42 | 0.03 | 0.42 | 0.95 | 0.0003 | 0.0007 | 0.66 | 0.22 |

A p-value of 0.05 was considered significant

Tertile 1,2 and 3 represent respectively the lowest, medium and highest milk consumers.

P for trend is the p for trend over non-consumers and all three tertiles
