# Supplementary material for: The association of dairy intake of children and adolescents with different food and nutrient intakes in the Netherlands
Source: BMC Pediatr. 2016 Jan 9;16:2. doi: 10.1186/s12887-015-0524-3 (PMC4707007; doi:10.1186/s12887-015-0524-3)
Supplement: Additional file 2: — Total nutrient intake over tertiles cheese consumption in children aged 7–13 years. A p-value of 0.05 was considered significant. Tertile 1,2 and 3 represent respectively the lowest, medium and highest cheese consumers. P for trend is the p for trend over non-consumers and all three tertiles. (DOCX 18 kb) [file 12887_2015_524_MOESM2_ESM.docx]

**Additional file 2. Total nutrient intake over tertiles cheese consumption in children aged 7-13 years**

|  | ***Non-cheese consumers*** | | ***Tertile 1*** | | ***Tertile 2*** | | ***Tertile 3*** | | | ***overall*** | | | |
| --- | --- | --- | --- | --- | --- | --- | --- | --- | --- | --- | --- | --- | --- |
| ***Per tertile cheese*** | ***estimate*** | ***St. error*** | ***estimate*** | ***St. error*** | ***estimate*** | ***St. error*** | ***estimate*** | ***St. error*** | ***p-value*** | ***estimate*** | ***St. error*** | ***p for trend*** | ***p for trend***  ***energy corrected*** |
| ***N*** | ***308*** |  | ***233*** |  | ***233*** |  | ***233*** |  |  | ***1007*** |  |  | ***1007*** |
| *Consumed quantity (g)* | *2078* | *28.0* | *-9.6* | *42.9* | *13.4* | *42.8* | *201* | *43.1* | *<.0001* | *0.32* | *0.07* | *<.0001* | *0.08* |
| *Energy (kcal)* | *2054* | *26.4* | *-45.4* | *40.5* | *2.8* | *40.3* | *203* | *40.6* | *<.0001* | *0.34* | *0.07* | *<.0001* | *xxx* |
| *Total protein(g)* | *62.7* | *1.06* | *-0.10* | *1.62* | *2.0* | *1.61* | *14.4* | *1.6* | *<.0001* | *0.02* | *0.003* | *<.0001* | *<.0001* |
| *Vegetable protein(g)* | *24.7* | *0.42* | *0.29* | *0.65* | *1.17* | *0.65* | *4.0* | *0.65* | *<.0001* | *0.007* | *0.001* | *<.0001* | *<.0001* |
| *Animal protein(g)* | *37.9* | *0.92* | *-0.42* | *1.41* | *0.89* | *1.41* | *10.4* | *1.42* | *<.0001* | *0.02* | *0.002* | *<.0001* | *<.0001* |
| *Total fat(g)* | *76.0* | *1.42* | *-0.11* | *2.2* | *0.61* | *2.2* | *10.8* | *2.2* | *<.0001* | *0.02* | *0.004* | *<.0001* | *0.37* |
| *Saturated fatty acids(g)* | *27.0* | *0.55* | *0.76* | *0.84* | *2.3* | *0.83* | *8.5* | *0.84* | *<.0001* | *0.01* | *0.001* | *<.0001* | *<.0001* |
| *Mono-unsaturated fatty acids cis(g)* | *27.9* | *0.56* | *-0.45* | *0.85* | *-0.93* | *0.85* | *1.63* | *0.86* | *0.06* | *0.002* | *0.001* | *0.10* | *0.0001* |
| *Poly-unsaturated fatty acids(g)* | *15.1* | *0.35* | *-0.46* | *0.53* | *-1.01* | *0.53* | *-0.42* | *0.53* | *0.44* | *-0.0009* | *0.86* | *0.30* | *<.0001* |
| *Trans fatty acids(g)* | *1.11* | *0.04* | *-0.01* | *0.06* | *0.17* | *0.06* | *0.48* | *0.06* | *<.0001* | *0.0008* | *0.10* | *<.0001* | *<.0001* |
| *N-3 fish fatty acids (EPA+DHA.mg)* | *82.1* | *11.8* | *-24.6* | *18.1* | *7.0* | *18.0* | *-20.2* | *18.1* | *0.27* | *-0.01* | *0.03* | *0.65* | *0.44* |
| *Total carbohydrates(g)* | *271* | *3.6* | *-11.2* | *5.6* | *-2.9* | *5.6* | *10.8* | *5.6* | *0.05* | *0.02* | *0.01* | *0.02* | *<.0001* |
| *Mono- and disaccharides(g)* | *151* | *2.6* | *-12.9* | *4.0* | *-6.6* | *4.0* | *-4.7* | *4.0* | *0.24* | *-0.003* | *0.01* | *0.61* | *<.0001* |
| *Polysaccharides(g)* | *120* | *1.84* | *1.67* | *2.8* | *3.7* | *2.8* | *15.5* | *2.8* | *<.0001* | *0.02* | *0.005* | *<.0001* | *0.02* |
| *Fibre(g)* | *16.0* | *0.27* | *0.09* | *0.42* | *-0.004* | *0.42* | *1.49* | *0.42* | *0.00* | *0.002* | *0.68* | *0.001* | *0.62* |
| *Alcohol(g)* | *0.01* | *0.02* | *0.06* | *0.03* | *-0.0005* | *0.03* | *0.03* | *0.03* | *0.32* | *0.000002* | *0.04* | *0.95* | *0.98* |
| *Calcium(mg)* | *769* | *19.0* | *27.3* | *29.0* | *133* | *28.9* | *454* | *29.1* | *<.0001* | *0.74* | *0.05* | *<.0001* | *<.0001* |
| *Copper(mg)* | *0.95* | *0.02* | *-0.01* | *0.02* | *-0.03* | *0.02* | *0.04* | *0.02* | *010* | *0.00005* | *0.04* | *0.20* | *0.001* |
| *Iron(mg)* | *8.5* | *0.14* | *-0.34* | *0.22* | *-0.21* | *0.22* | *0.37* | *0.22* | *0.088* | *0.001* | *0.35* | *0.07* | *0.04* |
| *Folate equivalents(µg)* | *177* | *4.4* | *-7.0* | *6.8* | *8.4* | *6.7* | *35.3* | *6.8* | *<.0001* | *0.06* | *0.01* | *<.0001* | *<.0001* |
| *Iodine(µg)* | *141* | *2.9* | *-3.3* | *4.4* | *6.2* | *4.4* | *32.0* | *4.5* | *<.0001* | *0.05* | *0.01* | *<.0001* | *<.0001* |
| *Potassium(mg)* | *2587* | *41.6* | *-90.8* | *63.7* | *-109* | *63.6* | *59.8* | *64.0* | *0.35* | *0.09* | *0.10* | *0.40* | *0.0002* |
| *Magnesium(mg)* | *247* | *4.0* | *-3.8* | *6.2* | *-3.1* | *6.2* | *26.1* | *6.2* | *<.0001* | *0.04* | *0.01* | *<.0001* | *0.37* |
| *Sodium(mg)* | *2107* | *38.6* | *83.9* | *59.1* | *162* | *59.0* | *615* | *59.3* | *<.0001* | *0.97* | *0.10* | *<.0001* | *<.0001* |
| *Phosphorus(mg)* | *1159* | *19.2* | *-2.4* | *29.4* | *56.6* | *29.4* | *318* | *29.5* | *<.0001* | *0.51* | *0.05* | *<.0001* | *<.0001* |
| *Selenium(µg)* | *33.1* | *0.72* | *0.12* | *1.10* | *0.19* | *1.10* | *5.5* | *1.10* | *.0001* | *0.01* | *0.002* | *<.0001* | *0.02* |
| *Zinc(mg)* | *7.6* | *0.16* | *0.22* | *0.24* | *0.63* | *0.24* | *2.3* | *0.24* | *<.0001* | *0.004* | *0.39* | *<.0001* | *<.0001* |
| *Retinol activity equivalents(µg)* | *564* | *28.9* | *49.4* | *44.2* | *93.1* | *44.1* | *178* | *44.4* | *<.0001* | *0.29* | *0.07* | *<.0001* | *0.01* |
| *Vitamin B1(mg)* | *0.97* | *0.03* | *-0.04* | *0.04* | *0.02* | *0.04* | *0.05* | *0.04* | *0.27* | *0.0001* | *0.07* | *0.12* | *0.52* |
| *Vitamin B2(mg)* | *1.37* | *0.03* | *-0.08* | *0.05* | *0.01* | *0.05* | *0.22* | *0.05* | *<.0001* | *0.0004* | *0.08* | *<.0001* | *0.01* |
| *Vitamin B6(mg)* | *1.63* | *0.05* | *-0.09* | *0.07* | *-0.10* | *0.07* | *0.09* | *0.07* | *0.22* | *0.0001* | *0.12* | *0.23* | *0.44* |
| *Vitamin B12(µg)* | *3.3* | *0.11* | *-0.15* | *0.16* | *0.13* | *0.16* | *0.9* | *0.16* | *<.0001* | *0.002* | *0.26* | *<.0001* | *<.0001* |
| *Vitamin C(mg)* | *82* | *2.7* | *-0.8* | *4.2* | *1.9* | *4.2* | *2.0* | *4.2* | *0.63* | *0.00* | *0.01* | *0.50* | *0.51* |
| *Vitamin D(µg)* | *2.6* | *0.08* | *-0.28* | *0.13* | *-0.15* | *0.13* | *0.05* | *0.13* | *0.70* | *0.0002* | *0.21* | *0.46* | *0.10* |
| *Vitamin E(mg)* | *11.3* | *0.28* | *-0.36* | *0.42* | *0.00* | *0.42* | *0.43* | *0.42* | *0.31* | *0.0009* | *0.68* | *0.21* | *0.13* |

A p-value of 0.05 was considered significant

Tertile 1,2 and 3 represent respectively the lowest, medium and highest cheese consumers.

P for trend is the p for trend over non-consumers and all three tertiles
