# Supplementary material for: The association of dairy intake of children and adolescents with different food and nutrient intakes in the Netherlands
Source: BMC Pediatr. 2016 Jan 9;16:2. doi: 10.1186/s12887-015-0524-3 (PMC4707007; doi:10.1186/s12887-015-0524-3)
Supplement: Additional file 3: — Total nutrient intake over tertiles milk consumption in children aged 14–18 years. A p-value of 0.05 was considered significant. Tertile 1,2 and 3 represent respectively the lowest, medium and highest milk consumers. P for trend is the p for trend over non-consumers and all three tertiles. (DOCX 22 kb) [file 12887_2015_524_MOESM3_ESM.docx]

**Additional file 3. Total nutrient intake over tertiles milk consumption in children aged 14-18 years**

|  | **Non-milk consumers** | | **Tertile 1** | | **Tertile 2** | | **Tertile 3** | | | **overall** | | | |
| --- | --- | --- | --- | --- | --- | --- | --- | --- | --- | --- | --- | --- | --- |
| **Per tertile milk** | **estimate** | **St. error** | **estimate** | **St. error** | **estimate** | **St. error** | **estimate** | **St. error** | **p-value** | **estimate** | **St. error** | **p for trend** | **p for trend**  **energy corrected** |
| **N** | **231** |  | **160** |  | **157** |  | **158** |  |  | **706** |  |  | **706** |
| Consumed quantity (g) | 2578 | 52.2 | 40 | 82 | 88 | 83 | 267 | 81.2 | 0.00 | 0.63 | 0.19 | 0.001 | 0.11 |
| Energy (kcal) | 2290 | 48.9 | 34 | 77 | 30 | 78 | 268 | 76.1 | 0.00 | 0.60 | 0.18 | 0.001 | <.0001 |
| Total protein(g) | 72.5 | 1.57 | 1.25 | 2.5 | 7.3 | 2.5 | 18.5 | 2.4 | <.0001 | 0.05 | 0.006 | <.0001 | <.0001 |
| Vegetable protein(g) | 31.0 | 0.72 | -0.88 | 1.13 | -0.31 | 1.14 | 2.9 | 1.11 | 0.01 | 0.007 | 0.003 | 0.01 | 0.90 |
| Animal protein(g) | 41.3 | 1.23 | 2.2 | 1.94 | 7.8 | 1.95 | 15.8 | 1.91 | <.0001 | 0.04 | 0.005 | <.0001 | <.0001 |
| Total fat(g) | 85.7 | 2.3 | 2.2 | 3.7 | 1.1 | 3.7 | 11.8 | 3.6 | 0.00 | 0.03 | 0.009 | 0.003 | 0.95 |
| Saturated fatty acids(g) | 30.9 | 0.86 | 1.19 | 1.36 | 1.26 | 1.37 | 7.3 | 1.34 | <.0001 | 0.02 | 0.003 | <.0001 | <.0001 |
| Mono-unsaturated fatty acids cis(g) | 30.8 | 0.93 | 0.92 | 1.46 | -0.10 | 1.47 | 2.81 | 1.44 | 0.05 | 0.006 | 0.003 | 0.10 | 0.02 |
| Poly-unsaturated fatty acids(g) | 17.0 | 0.57 | -0.04 | 0.90 | -0.14 | 0.91 | 0.74 | 0.89 | 0.40 | 0.002 | 0.002 | 0.45 | 0.01 |
| Trans fatty acids(g) | 1.34 | 0.05 | 0.02 | 0.08 | -0.02 | 0.08 | 0.18 | 0.08 | 0.03 | 0.0004 | 0.0002 | 0.05 | 0.76 |
| N-3 fish fatty acids (EPA+DHA.mg) | 62.4 | 15.9 | 19.2 | 25.2 | 37.9 | 25.4 | 31.4 | 15.6 | 0.05 | 0.12 | 0.06 | 0.04 | 0.04 |
| Total carbohydrates(g) | 287 | 6.0 | 5.0 | 9.5 | -1.10 | 9.5 | 21.3 | 9.3 | 0.02 | 0.04 | 0.02 | 0.05 | 0.03 |
| Mono- and disaccharides(g) | 143 | 3.8 | 5.4 | 6.1 | -5.3 | 6.1 | 11.3 | 6.0 | 0.06 | 0.018 | 0.01 | 0.20 | 0.18 |
| Polysaccharides(g) | 144 | 3.2 | -0.40 | 5.1 | 4.1 | 5.2 | 10.0 | 5.0 | 0.05 | 0.03 | 0.01 | 0.03 | 0.19 |
| Fibre(g) | 18.9 | 0.45 | 0.59 | 0.71 | 0.51 | 0.72 | 1.63 | 0.70 | 0.02 | 0.004 | 0.002 | 0.03 | 0.90 |
| Alcohol(g) | 5.2 | 1.25 | -1.71 | 1.98 | -0.85 | 1.99 | -0.19 | 1.95 | 0.92 | 0.0002 | 0.005 | 0.96 | 0.17 |
| Calcium(mg) | 752 | 25.1 | 109 | 39.5 | 251 | 39.9 | 664 | 39.0 | <.0001 | 1.58 | 0.09 | <.0001 | <.0001 |
| Copper(mg) | 1.11 | 0.03 | 0.01 | 0.04 | 0.00 | 0.04 | 0.11 | 0.04 | 0.01 | 0.0002 | 0.00009 | 0.01 | 0.98 |
| Iron(mg) | 9.2 | 0.21 | 0.44 | 0.33 | 0.54 | 0.33 | 1.01 | 0.33 | 0.002 | 0.002 | 0.0008 | 0.003 | 0.30 |
| Folate equivalents(µg) | 202 | 6.4 | 17.8 | 10.0 | 30.4 | 10.1 | 62.2 | 9.9 | <.0001 | 0.15 | 0.02 | <.0001 | <.0001 |
| Iodine(µg) | 161 | 4.1 | -6.7 | 6.4 | 9.6 | 6.5 | 46.1 | 6.3 | <.0001 | 0.11 | 0.02 | <.0001 | <.0001 |
| Potassium(mg) | 2610 | 60.2 | 253 | 95.1 | 441 | 95.8 | 924 | 93.7 | <.0001 | 2.2 | 0.22 | <.0001 | <.0001 |
| Magnesium(mg) | 275 | 6.6 | 11.4 | 10.5 | 29.0 | 10.5 | 76.2 | 10.3 | <.0001 | 0.18 | 0.02 | <.0001 | <.0001 |
| Sodium(mg) | 2611 | 60.2 | 34.5 | 95.1 | 99.1 | 95.8 | 290 | 93.7 | 0.00 | 0.69 | 0.22 | 0.00 | 0.33 |
| Phosphorus(mg) | 1271 | 29.8 | 72.5 | 47.0 | 218 | 47.4 | 561 | 46.3 | <.0001 | 1.36 | 0.11 | <.0001 | <.0001 |
| Selenium(µg) | 40 | 1.01 | -0.01 | 1.59 | 2.4 | 1.60 | 4.8 | 1.56 | 0.002 | 0.01 | 0.004 | 0.001 | 0.07 |
| Zinc(mg) | 9.1 | 0.22 | 0.22 | 0.35 | 0.65 | 0.35 | 2.4 | 0.34 | <.0001 | 0.006 | 0.0008 | <.0001 | <.0001 |
| Retinol activity equivalents(µg) | 591 | 51.2 | 194 | 80.8 | 36.9 | 81.5 | 221 | 79.7 | 0.006 | 0.39 | 0.19 | 0.04 | 0.16 |
| Vitamin B1(mg) | 1.05 | 0.04 | 0.01 | 0.06 | 0.13 | 0.06 | 0.14 | 0.06 | 0.02 | 0.0004 | 0.0001 | 0.00 | 0.06 |
| Vitamin B2(mg) | 1.22 | 0.05 | 0.19 | 0.07 | 0.37 | 0.07 | 0.89 | 0.07 | <.0001 | 0.002 | 0.0002 | <.0001 | <.0001 |
| Vitamin B6(mg) | 1.97 | 0.07 | 0.01 | 0.11 | 0.11 | 0.12 | 0.05 | 0.11 | 0.69 | 0.0002 | 0.0003 | 0.52 | 0.39 |
| Vitamin B12(µg) | 3.1 | 0.14 | 0.53 | 0.23 | 0.93 | 0.23 | 2.4 | 0.22 | <.0001 | 0.006 | 0.0005 | <.0001 | <.0001 |
| Vitamin C(mg) | 95 | 4.0 | 6.5 | 6.3 | -6.5 | 6.4 | 6.2 | 6.2 | 0.32 | 0.005 | 0.01 | 0.74 | 0.86 |
| Vitamin D(µg) | 2.7 | 0.12 | -0.01 | 0.18 | 0.36 | 0.18 | 0.15 | 0.18 | 0.40 | 0.0006 | 0.0004 | 0.17 | 0.98 |
| Vitamin E(mg) | 13.4 | 0.49 | -0.27 | 0.77 | -0.37 | 0.77 | -0.50 | 0.76 | 0.51 | -0.001 | 0.002 | 0.51 | 0.00 |

A p-value of 0.05 was considered significant

Tertile 1,2 and 3 represent respectively the lowest, medium and highest milk consumers.

P for trend is the p for trend over non-consumers and all three tertiles
