# Supplementary material for: The association of dairy intake of children and adolescents with different food and nutrient intakes in the Netherlands
Source: BMC Pediatr. 2016 Jan 9;16:2. doi: 10.1186/s12887-015-0524-3 (PMC4707007; doi:10.1186/s12887-015-0524-3)
Supplement: Additional file 4: — Total nutrient intake over tertiles cheese consumption in children aged 14–18 years. A p-value of 0.05 was considered significant. Tertile 1,2 and 3 represent respectively the lowest, medium and highest cheese consumers. P for trend is the p for trend over non-consumers and all three tertiles. (DOCX 19 kb) [file 12887_2015_524_MOESM4_ESM.docx]

**Additional file 4. Total nutrient intake over tertiles cheese consumption in children aged 14-18 years**

|  | **Non-cheese consumers** | | **Tertile 1** | | **Tertile 2** | | **Tertile 3** | | | **overall** | | | |
| --- | --- | --- | --- | --- | --- | --- | --- | --- | --- | --- | --- | --- | --- |
| **Per tertile cheese** | **estimate** | **St. error** | **estimate** | **St. error** | **estimate** | **St. error** | **estimate** | **St. error** | **p-value** | **estimate** | **St. error** | **p for trend** | **p for trend**  **energy corrected** |
| **N** | **192** |  | **165** |  | **179** |  | **170** |  |  | **706** |  |  | **706** |
| Consumed quantity (g) | 2541 | 57.4 | 0.38 | 84.4 | 158 | 82.2 | 354 | 83.2 | <.0001 | 0.85 | 0.18 | <.0001 | 0.01 |
| Energy (kcal) | 2283 | 53.8 | -37.2 | 79.1 | 42.0 | 77.0 | 331 | 77.9 | <.0001 | 0.77 | 0.17 | <.0001 | xx |
| Total protein(g) | 73.5 | 1.77 | 0.62 | 2.6 | 5.2 | 2.5 | 15.4 | 2.6 | <.0001 | 0.04 | 0.006 | <.0001 | <.0001 |
| Vegetable protein(g) | 30.4 | 0.79 | -0.89 | 1.16 | 0.56 | 1.13 | 4.6 | 1.14 | <.0001 | 0.01 | 0.002 | <.0001 | 0.12 |
| Animal protein(g) | 42.9 | 1.40 | 1.60 | 2.1 | 4.7 | 2.0 | 10.8 | 2.0 | <.0001 | 0.02 | 0.004 | <.0001 | 0.0002 |
| Total fat(g) | 83.5 | 2.6 | 0.27 | 3.8 | 6.2 | 3.7 | 16.3 | 3.7 | <.0001 | 0.04 | 0.008 | <.0001 | 0.12 |
| Saturated fatty acids(g) | 28.1 | 0.91 | 2.3 | 1.34 | 5.4 | 1.31 | 12.5 | 1.32 | <.0001 | 0.03 | 0.003 | <.0001 | <.0001 |
| Mono-unsaturated fatty acids cis(g) | 31.1 | 1.02 | -1.14 | 1.51 | 0.37 | 1.47 | 3.0 | 1.48 | 0.047 | 0.008 | 0.003 | 0.02 | 0.004 |
| Poly-unsaturated fatty acids(g) | 17.3 | 0.63 | -0.75 | 0.93 | 0.03 | 0.90 | -0.24 | 0.92 | 0.79 | 0.0002 | 0.002 | 0.93 | <.0001 |
| Trans fatty acids(g) | 1.20 | 0.06 | 0.07 | 0.08 | 0.20 | 0.08 | 0.48 | 0.08 | <.0001 | 0.001 | 0.18 | <.0001 | <.0001 |
| N-3 fish fatty acids (EPA+DHA.mg) | 101 | 17.7 | -15.3 | 26.0 | -4.4 | 25.3 | -42.3 | 25.6 | 0.10 | -0.08 | 0.06 | 0.15 | 0.13 |
| Total carbohydrates(g) | 294 | 6.6 | -14.4 | 9.7 | -8.9 | 9.5 | 19.9 | 9.6 | 0.04 | 0.05 | 0.02 | 0.02 | 0.0002 |
| Mono- and disaccharides(g) | 150 | 4.3 | -9.5 | 6.3 | -9.6 | 6.1 | 0.8 | 6.2 | 0.90 | 0.004 | 0.01 | 0.74 | 0.0002 |
| Polysaccharides(g) | 143 | 3.5 | -4.8 | 5.2 | 0.75 | 5.1 | 19.2 | 5.1 | 0.0002 | 0.05 | 0.01 | <.0001 | 0.64 |
| Fibre(g) | 18.5 | 0.50 | 0.28 | 0.73 | 1.14 | 0.71 | 2.5 | 0.72 | 0.001 | 0.006 | 0.002 | 0.000 | 0.38 |
| Alcohol(g) | 3.0 | 1.38 | 2.0 | 2.0 | -0.29 | 1.98 | 5.00 | 2.00 | 0.01 | 0.009 | 0.004 | 0.04 | 0.77 |
| Calcium(mg) | 753 | 29.5 | 121 | 43.4 | 249 | 42.3 | 573 | 42.8 | <.0001 | 1.27 | 0.09 | <.0001 | <.0001 |
| Copper(mg) | 1.12 | 0.03 | -0.04 | 0.04 | 0.01 | 0.04 | 0.08 | 0.04 | 0.053 | 0.0002 | 0.09 | 0.02 | 0.10 |
| Iron(mg) | 9.5 | 0.23 | -0.18 | 0.34 | 0.38 | 0.33 | 0.65 | 0.34 | 0.056 | 0.002 | 0.73 | 0.02 | 0.37 |
| Folate equivalents(µg) | 206 | 7.1 | 1.55 | 10.4 | 28.4 | 10.1 | 55.6 | 10.2 | <.0001 | 0.14 | 0.02 | <.0001 | <.0001 |
| Iodine(µg) | 162 | 4.6 | -5.3 | 6.8 | 13.1 | 6.6 | 30.8 | 6.7 | <.0001 | 0.08 | 0.01 | <.0001 | 0.001 |
| Potassium(mg) | 2917 | 71.0 | -63.0 | 104 | 74 | 102 | 224 | 103 | 0.03 | 0.58 | 0.22 | 0.01 | 0.21 |
| Magnesium(mg) | 290 | 7.6 | 0.98 | 11.1 | 8.1 | 10.8 | 40.6 | 11.0 | 0.0002 | 0.09 | 0.02 | 0.00 | 0.52 |
| Sodium(mg) | 2569 | 64.6 | -152 | 94.9 | 150 | 92.5 | 556 | 93.6 | <.0001 | 1.42 | 0.20 | <.0001 | <.0001 |
| Phosphorus(mg) | 1315 | 34.8 | 51.6 | 51.1 | 148 | 49.8 | 410 | 50.4 | <.0001 | 0.92 | 0.11 | <.0001 | <.0001 |
| Selenium(µg) | 40.1 | 1.11 | -1.19 | 1.64 | 1.43 | 1.59 | 4.6 | 1.61 | 0.004 | 0.01 | 0.003 | 0.001 | 0.29 |
| Zinc(mg) | 9.2 | 0.24 | 0.04 | 0.36 | 0.80 | 0.35 | 2.2 | 0.35 | <.0001 | 0.005 | 0.77 | <.0001 | <.0001 |
| Retinol activity equivalents(µg) | 609 | 57.0 | 22.7 | 83.7 | 132 | 81.6 | 186 | 82.5 | 0.02 | 0.46 | 0.18 | 0.0105 | 0.09 |
| Vitamin B1(mg) | 1.16 | 0.04 | -0.11 | 0.06 | -0.07 | 0.06 | -0.05 | 0.06 | 0.39 | -0.0001 | 0.13 | 0.69 | 0.03 |
| Vitamin B2(mg) | 1.47 | 0.06 | -0.03 | 0.08 | 0.08 | 0.08 | 0.28 | 0.08 | 0.001 | 0.0007 | 0.18 | 0.00 | 0.06 |
| Vitamin B6(mg) | 2.1 | 0.08 | -0.20 | 0.12 | -0.05 | 0.12 | 0.02 | 0.12 | 0.83 | 0.0002 | 0.25 | 0.42 | 0.21 |
| Vitamin B12(µg) | 3.6 | 0.17 | -0.04 | 0.25 | 0.59 | 0.24 | 0.98 | 0.25 | <.0001 | 0.002 | 0.53 | <.0001 | 0.001 |
| Vitamin C(mg) | 96 | 4.4 | -4.1 | 6.5 | -1.21 | 6.4 | 5.7 | 6.4 | 0.38 | 0.02 | 0.01 | 0.28 | 0.69 |
| Vitamin D(µg) | 2.9 | 0.13 | -0.29 | 0.19 | -0.01 | 0.18 | 0.08 | 0.19 | 0.68 | 0.0004 | 0.40 | 0.30 | 0.36 |
| Vitamin E(mg) | 13.1 | 0.54 | -0.95 | 0.79 | 0.001 | 0.77 | 1.14 | 0.78 | 0.14 | 0.003 | 0.002 | 0.05 | 0.46 |

A p-value of 0.05 was considered significant

Tertile 1,2 and 3 represent respectively the lowest, medium and highest cheese consumers.

P for trend is the p for trend over non-consumers and all three tertiles
